# Dynamic Sensorimotor Planning during Long-Term Sequence Learning: The Role of Variability, Response Chunking and Planning Errors

**DOI:** 10.1371/journal.pone.0047336

**Published:** 2012-10-09

**Authors:** Timothy Verstynen, Jeff Phillips, Emily Braun, Brett Workman, Christian Schunn, Walter Schneider

**Affiliations:** 1 Learning Research and Development Center, University of Pittsburgh, Pittsburgh, Pennsylvania, United States of America; 2 Center for the Neural Basis of Cognition, University of Pittsburgh, Pittsburgh, Pennsylvania, United States of America; McMaster University, Canada

## Abstract

Many everyday skills are learned by binding otherwise independent actions into a unified sequence of responses across days or weeks of practice. Here we looked at how the dynamics of action planning and response binding change across such long timescales. Subjects (N = 23) were trained on a bimanual version of the serial reaction time task (32-item sequence) for two weeks (10 days total). Response times and accuracy both showed improvement with time, but appeared to be learned at different rates. Changes in response speed across training were associated with dynamic changes in response time variability, with faster learners expanding their variability during the early training days and then contracting response variability late in training. Using a novel measure of response chunking, we found that individual responses became temporally correlated across trials and asymptoted to set sizes of approximately 7 bound responses at the end of the first week of training. Finally, we used a state-space model of the response planning process to look at how predictive (i.e., response anticipation) and error-corrective (i.e., post-error slowing) processes correlated with learning rates for speed, accuracy and chunking. This analysis yielded non-monotonic association patterns between the state-space model parameters and learning rates, suggesting that different parts of the response planning process are relevant at different stages of long-term learning. These findings highlight the dynamic modulation of response speed, variability, accuracy and chunking as multiple movements become bound together into a larger set of responses during sequence learning.

## Introduction

Many complex skills involve learning to bind discrete, independent actions into a unified sequence of responses. For example, consider a novice piano student trying to learn *Fur Elise* for the first time. Mastering this simple melody requires learning to bind many independent hand movements into a well-timed and unified sequence of actions. After an hour, the student might be able to pick up small parts of the melody, but mastering the overall song requires days or weeks of practice.

This sequential learning is a multifaceted process, with both implicit procedural and explicit conscious components [1]–[4]. For example, when imperative cues for an action, e.g., key presses, are presented in a sequential fashion, the speed and accuracy of the actions steadily improve with practice [4] even if subjects are not explicitly aware of the cue ordering [5]. In fact these implicit and explicit components appear to be consolidated differently [6], illustrating the role of multiple plasticity mechanisms during sequential skill learning. The transition from implicit (early) to explicit (late) learning is thought to reflect different stages of the long-term consolidation [1], [3], [6] and likely reflects the recruitment of different neural systems at different stages of learning [2], [3], [7]–[12].

During the explicit stages of learning, performance changes are accelerated compared to changes observed in implicit stages. This acceleration with explicit awareness is thought to result, in part, from associative processes that facilitate identification of relational patterns between items in the cued sequence [1], [13]–[15]. This binding of responses is sometimes referred to as “chunking” [13], [16]–[26]. Of the hundreds of studies on manual sequence learning in humans, only a small subset have focused on the process of response binding itself. In a typical “chunking” study, a simple (i.e., 3-12 item) sequence is used and the repetition structure of elements in the sequence is manipulated (e.g., “abcabc” vs. “dacbdc”). With practice, the first item in the concatenated set of actions exhibits a slower response time (RT) than the rest of the elements in the set. This slowing is used as an index of the segmentation of the learned chunk [12], [13], [16]–[22], [25]–[28].

Several lines of evidence suggest this binding of actions may happen during the response planning stages or higher executive decision-making states: (1) chunking is correlated with working memory capacity, but not simple motor production abilities [27], [29], [30], (2) it is context-specific (i.e., chunks of one sequence do not transfer to another sequence with similar structure [16]), (3) the structure of chunked responses is not affected by manipulations of execution parameters (e.g., target distance, effector)[13]. Although these findings highlight the high level cognitive processes that may mediate response binding, the simplicity of the sequences being tested limit our understanding of the capacity of this aspect of sequence learning.

In addition, most of the behavioral sequence learning studies in humans have focused on learning-related changes that occur within, at most, a few days of training. However, outside the laboratory, complex skills are acquired over the course of days, weeks or months of training. Indeed, this long timescale of learning is supported by functional imaging studies showing a transition from primarily cortical systems early in learning to subcortical systems, like the basal ganglia, after several days or weeks of training [2], [7]–[9], [11], [28], [31]. This long timescale is also supported by studies in non-human animals that also find a long timescale of consolidation for skill learning [23], [24], [32], [33].

When interpreting behavioral dynamics in sequence learning, it is generally assumed that the properties of sensorimotor planning are the same at both short (i.e., 1-2 days) and long (i.e., weeks or months) timescales of training. However, there is evidence that the dynamics of sensorimotor planning do not, in fact, change monotonically over time during sequence learning, but are instead modulated over the course of long-term training. In songbirds, infant and juvenile birds learn to acquire the sequential production of “syllables” in social calls by emulating an adult tutor (see [34] for review). During early learning, these sequences of calls, or “motifs”, are highly variable in both spectral and temporal structure. Over time these calls crystalize into a well learned and highly stable song, due in large part to structures analogous to the human cortico-basal ganglia system, called the anterior forebrain pathway (AFP)[35]. In the so-called “sensorimotor phase” of learning, juvenile birds modulate their songs depending on whether the call is directed at a conspecific female or not. During the undirected songs the sequential structure of the vocalization becomes more variable, as does the firing pattern of AFP cells tuned to the production of the bird's own song[36]–[38]. This dynamic modulation of response variability in undirected songs acts as a way of exploring the space of vocalizations in order to maximize learning and identify an optimal song structure most appealing to mates. Taken together with results from the human neuroimaging literature [2], [7]–[9], [11], [28], [31], these findings in the songbird support the hypothesis response planning systems change across the course of consolidation during extended practice.

Here we set out to describe the dynamics of learning in a sensorimotor sequence task that is trained across more ecological timescales of skill learning. We used a bimanual version of the classic serial reaction time (SRT) task [4], with a complex sequence that was designed to prolong the transition from implicit to explicit learning stages as well as evaluate the extent of response binding in a naturalistic way. During this training we measured behavioral responses, and modeled the underlying computational dynamics of response planning across a more ecologically valid time-scale of two weeks (10 training days). Our results reveal a complex interplay between movement, speed, variability and accuracy as independent movements become bound into a singular action plan.

## Methods

### Ethics Statement

All experimental procedures were approved by the local ethical review board at the University of Pittsburgh and all participants provided written informed consent to participate in the study.

### Participants

Twenty-five neurologically health adults (16 female, age  = 18–30 years, all right handed), were recruited from the University of Pittsburgh student population. All subjects were financially compensated for their time.

### Experimental Protocol

All participants were trained for 10 days (five weekdays for two weeks) on a bimanual SRT paradigm. On each day of training, participants were seated comfortably in front of a computer monitor. Both the left and right hand were placed on a 5 key response glove (PST Inc) and participants were allowed to arrange these gloves on the table in front of them to maximize comfort and ease of responding.

**Figure 1:**
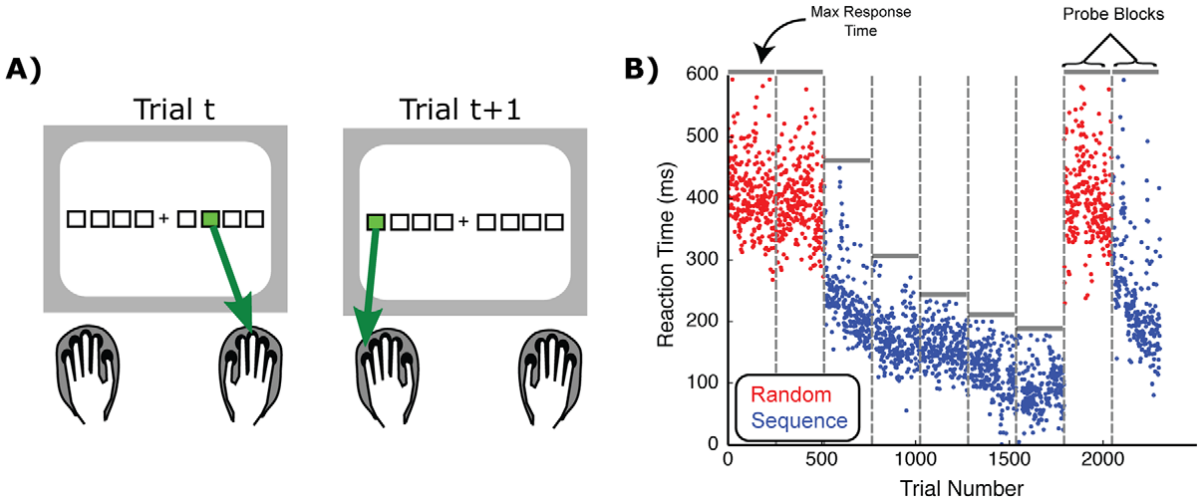
The bimanual Serial Reaction Time task. A) Subjects saw eight response cues on a computer screen that were spatially aligned with each non-thumb finger. Imperative cues (green box) were presented one at a time on each trial and subjects were cued to press the corresponding key as quickly as possible. B) Example training structure and reaction times from a representative subject (tenth training session). Each dot represents a single trials reaction time and the vertical bars indicate the breaks between blocks. Horizontal lines within each block show the maximum response window. See text for experimental details.

All stimulus presentation and behavioral recording was performed using EPrime2 software (PST Inc). During each trial, eight response cues were spatially arrayed on the screen. These cues consisted of white boxes, with four placed to the left of a fixation cross and four placed to the right (Figure 1a). The fixation cross was not used to restrict eye movements but only to spatially separate the cues for the two hands. Each box spatially corresponded to a key on the response pad (thumbs were excluded for responses). For example the left-most box corresponded to the left pinky key and the right-most box corresponded to the right pinky key. On each trial, a single box would turn green (“imperative cue”) to indicate that the participant should press the corresponding key. Participants were given no instruction other than to press the key as quickly as possible. If subjects pressed an incorrect key, all eight boxes flashed red for 200 ms to provide feedback of the error. There was also a 200 ms interval between the last response and the following trial cue.

Trial blocks (256 trials per block) were broken into two types of trials. During Sequence blocks, the trial-by-trial order of the imperative cue followed a preset 32-item sequence of 6-8-5-6-3-5-4-1-3-6-8-4-1-2-7-3-1-8-2-7-5-2-4-5-7-3-1-6-8-2-4-7, using the following key mapping: 1 = left pinky, 2 =  left ring finger, 3 =  left middle finger, 4 =  left index finger, 5 =  right index finger, 6 =  right middle finger, 7 =  right ring finger, and 8 =  right pinky finger. The sequence structure was explicitly designed to avoid any “triplet” structures that could aid in the detection and learning of the pattern [14]. At the beginning of each Sequence block, the first cue would start at a random position along the sequence in order to reduce detection of the sequence from the first few trials. During each Sequence block the entire cue sequence was repeated eight times. In the Random condition, the trial-by-trial ordering of the cue was presented in a pseudo-random order. This was pseudo-random because repeated presentations of the same cue were eliminated so as to make the cue presentation appear as similar as possible to the Sequence condition. After each block of trials, participants were given feedback on their mean reaction time and accuracy. Participants were allowed to continue to the next block at their own pace.

Each training day was divided into nine testing blocks (Figure 1b). The first two blocks were Random trials. During these trials, participants had a maximum response window of 600 ms. If the response was longer than this time, an error signal was presented to the subject (see above). The next five trial blocks were adaptive Sequence blocks that reflected the core training period. These blocks were adaptive because the response window was shortened on each block based on the mean (µ_RT_) and standard deviation (σ_RT_) of the reaction times during the previous block: maximum response time  =  µ_RT_ + σ_RT_. If this value fell below 200 ms or the accuracy on the previous block fell below 75% correct, this window was reset to the 600 ms default. After the adaptive training blocks, two probe blocks were presented to test learning related effects in the absence of the adaptive response window. The first probe was a Random condition, with a 600 ms maximum response window. The second probe, and last trial block, was a Sequence condition with a 600 ms maximum response window.

After each day of training, participants were verbally given a post-hoc questionnaire by one of the experimenters designed to assess their explicit awareness of the presence of the sequence. This questionnaire consisted of four questions. First, participants were asked “Did any of the stimuli appear different than the other?” Participants who answered “no” were given a score of 0 for that day and sent home. Participants who answered “yes” were then asked the second question “If so, in what way?” If subjects failed to respond with any one of the following key words they were given a score of 1 and sent home for the day: “pattern”, “sequence”, “sequential”, “order”, or “ordering”. If subjects responded with one of the key terms, they then were then asked “Was the pattern always present or just occasionally?” If participants responded that the pattern was “always present”, then they were given a score of 2 and sent home. If participants responded that the sequence was only occasionally present, then they were asked the fourth and final question, “Can you replicate any part of the sequence now?” If the subject could accurately reproduce at least 4 consecutive items in the sequence by visually showing the experimenter the movements or verbally recalling them, then the subject was given a final score of 3.

### Data Analysis

All data analysis was restricted to the last two probe blocks (one Random and one Sequence). The time series of reaction times and vector of correct/incorrect responses for each trial were extracted on each day from the two probe blocks separately. For time-series analysis, missing reaction time values (i.e., response time outside the maximum response window) were replaced with the mean value, otherwise these trials were excluded from analysis. To control for general, non-sequence specific changes in response speed (e.g., improved simple reaction time), response times during the Sequence probe was measured relative to the distribution of reaction times in the Random probe block and reflected as a z-score: (µ_Random_ – µ_Sequence_)/ σ_Random_. Accuracy was determined by looking at the percent correct trials during the Sequence probe block.

To estimate the rate of learning across training days for each subject, we fit an ordinary least square regression model to the average day-by-day sequence-specific RTs and accuracy rates. Because some subjects may asymptote with learning, we fit two models: 1) a simple linear model (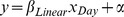), 2) and a quadratic model (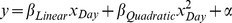). A likelihood ratio test was used to determine when the quadratic model provided a significantly better fit than the simple linear model. This also provides a direct test of asymptotic behavior in the learning rates. In cases where the linear model was the best fit, the subject's across-day learning score (λ) was estimated as: λ  =  β_Linear_. When the quadratic model was the better fit, the subject's across-day learning score was determined by summing the linear and quadratic components of the regression model: λ  =  β_Linear_ + β_Quadratic_.

Once the across-day learning measures were taken, we then looked at the influence of an error on subsequent trial responses. To do this we calculated the error response function (ERF) during each probe block, which is an estimate of the degree of post-error slowing [39]–[41], by taking the average reaction time of the subsequent six trials after an error (red vertical lines in Figure 3a). This number of post-error trials was determined based on pilot analysis showing no significant effects after 6 trials (see Figure 6). One ERF was calculated for each subject on each training day. Separate ERFs were calculated for the Random and Sequence probe blocks.

In order to look at the inter-trial dynamics of reaction times during both probe blocks, the first 32-trials (i.e., first sequence run) were excluded from analysis because these trials often exhibited an exponential decrease in reaction times during the Sequence probe block on later training days (see Figure 4a). In addition, the linear trend in subsequent trials was removed using an ordinary least squares linear regression approach and the time-series zero-mean. This vector of response times was then used to look at the inter-trial correlation (using *xcorr.m* in Matlab) across the entire length of the sequence, i.e., 31 lags. This autocorrelation function was estimated independently for the Random and Sequence probe blocks on each day for each subject.

With the autocorrelation of response times, we next estimated the size of a chunked set of responses by determining the number of significant, consecutive non-zero lags observed in the Sequence Probe block for each subject and each training day. On each day, the null distribution was estimated by taking the across-subject mean, µ(*l*), and standard deviation, σ(*l*),of the autocorrelation function, at each lag *l*, from responses in the Random Probe block. For each subject we then estimated a one-sample t-test for the autocorrelation value at each lag, ρ(*l*), as 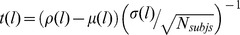. The number of consecutive, significant t-tests starting at lag = 1 was then as an estimate of sequence chunk size. Significance was estimated using a Bonferroni corrected alpha for 31 comparisons, i.e., the length of the autocorrelation function for each subject and each day.

### State-space Model

Finally, we modeled the trial-by-trial dynamics of response planning using a linear dynamical systems approach that has been described elsewhere [22], [23]. This state-space approach uses the expectation and maximization algorithm to fit the parameters of a model of the dynamics of an unmeasured internal state, *X_t_*, based on observable output values, *Y_t_*, and input values. In our case, the internal state reflects the preparedness to make a fast response on the next trial. We modeled the response preparedness dynamics for each Sequence probe block and each subject using the following equations:

### State Update



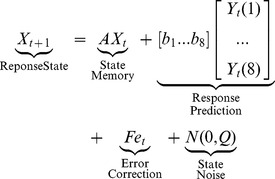
(1)


### Output



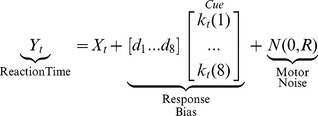
(2)On each trial, the reaction time, *Y_t_*, is a function of the unseen response preparedness state, *X_t_*, and the response bias, **D**, to each stimulus cue, **K**, plus motor noise, **R**. We modeled the response bias of each key separately so **D** is a 1x8 vector of response bias weights and **K** is a 8x1 binary vector where k_t_(i)  = 1 if the i^th^ cue is presented, otherwise k_t_(i)  = 0. After each trial, the internal response preparedness state is updated based on: 1) a degree of retention of the previous trial's state, A; 2) the influence of the reaction time on the previous trial, **B**; 3) the influence of an error, **F**, on the previous trial; 4) internal state noise, **Q**. In the state update, the **Y** vector is a binary vector indicating which key was pressed on the previous trial, regardless of its accuracy, and *e_t_* is a binary scalar that is 1 if the previous response was an error and 0 if it wasn't.

An expectation-maximization algorithm was used to estimate the free parameters **A**, **B**, **D**, **F**, **Q**, and **R** based on the observable vectors of reaction times, **Y**, and errors, **E**, from each trial [24]. In this way, the free parameters and the estimated internal state vector, **X**, are all in units of reaction times, i.e., milliseconds. Negative values for *X_t_* reflect trials where the participant is prepared to make a faster response than the mean (i.e., “prepared” trials), while positive values are trials where the response is delayed relative to the mean (i.e., “hesitation” trials).

## Results

### Response times and accuracy

**Figure 2:**
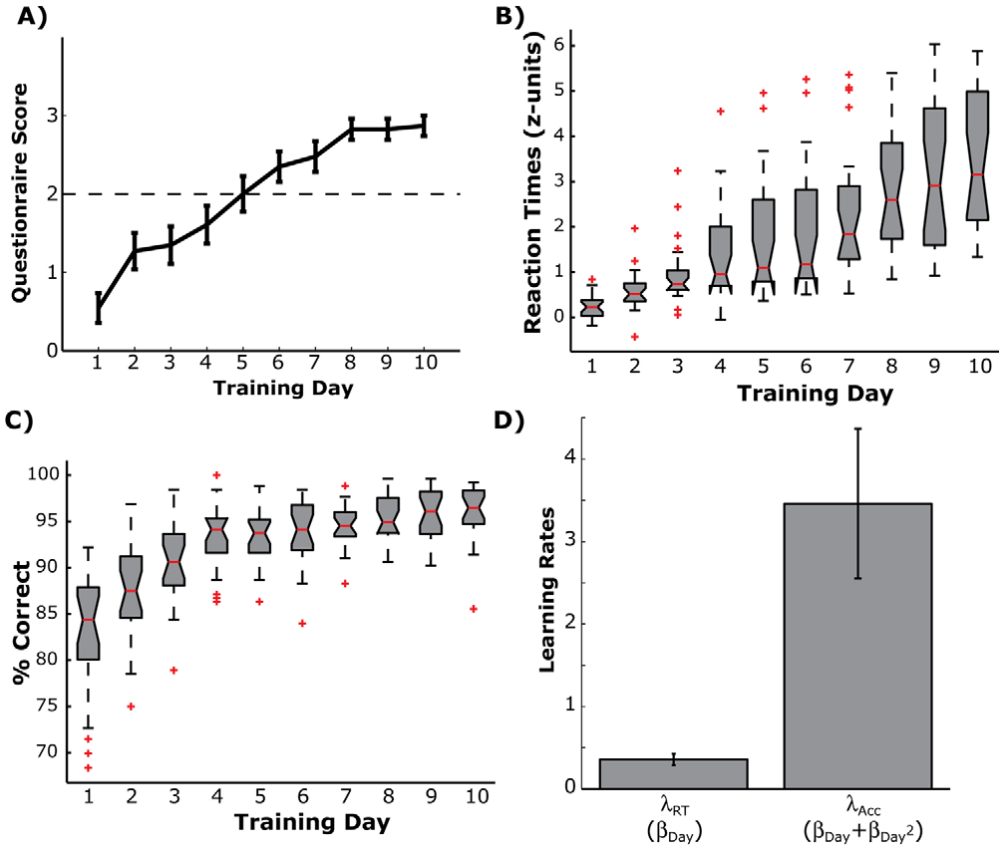
Performance changes across the training period. A) Mean and standard error of the report scores to the post-hoc questionnaire for each training day. Dashed line shows the explicit detection threshold. B) Whisker plots showing the distribution of response time changes for each day of training. The box edges show the upper and lower quartile ends (i.e., the 25^th^ and 75^th^ percentile), while the whisker lengths show the 90% confidence intervals and the red crosses show outliers beyond the confidence interval. The horizontal red line shows the median. C) Same plotting conventions as *B*, but for the percent correct trials during the Sequence probe block. D) Distribution of subject learning rates for response times (λ_RT_) and accuracy (λ_Acc_). Errorbars show the 95% confidence interval across subjects.

Two subjects were excluded from the final analysis for failure to complete all 10 days of training. The post-hoc questionnaires showed a steadily increasing awareness of the presence of the sequence across training days (Figure 2a; repeated measures F(22,207)  = 30.68, p<0.001). A score of 2 indicates transition from implicit to explicit detection of the sequence, since this is the point where participants are aware of the presence of a pattern on some blocks, but cannot explicitly relay a 4-item chunk. On average the group passed this awareness threshold after Day 5 of training.

Sequence-specific response times showed a steady improvement across all 10 training days (repeated measures F(22, 207)  = 59.39, p<0.001). Figure 2b shows the distribution of sequence-specific response time changes (see Methods, Data Analysis) across subjects for each training day. Learning does not appear to asymptote by the end of training. All but one subject (p = 0.034) failed to show a better fit with the quadratic model than the simple linear model; significance for that single subject disappears after adjusting for multiple comparisons (see Methods, Data Analysis for details). Therefore, individual subject across-day learning rates on response times (λ_RT_) were modeled with a simple linear equation.

Accuracy scores during the Sequence probe block also showed improvement across training (Figure 2c; repeated measures F(22, 207)  = 40.94, p<0.001). However, unlike response times, accuracy rates appeared to plateau after the fifth day of training, where participants performed at a constant 93–95% accuracy for the last week of training. Only one subject had a non-significant likelihood ratio test for the quadratic model (p = 0.174). For all other subjects, the across day learning rates on accuracy were better fit by a quadratic model than a simple linear model (all p's < 0.0017). Therefore, we chose to use the quadratic model to quantify each subject's rate of change of accuracy across training (λ_Acc_).

An inspection of the single-subject learning rates for both response time and accuracy (Figure 2d) reveals both highly significant learning at the group level and substantial inter-subject variability (λ_RT_  = 0.357 +/-0.167, λ_Acc_  = 3.46+/-2.22; mean +/- standard deviation). Despite the range of individual variability in learning rates, participants appeared to change their speed and their accuracy independently. While the direction of the correlation between λ_RT_ and λ_Acc_ was negative, as expected from a speed-accuracy trade-off, it did not reach statistical significance (Spearman's r  = −0.18, p = 0.18). This lack of correlation in the learning rates suggests that response speed and response accuracy are learned at independent rates in this sample.

### Response variability and learning

**Figure 3:**
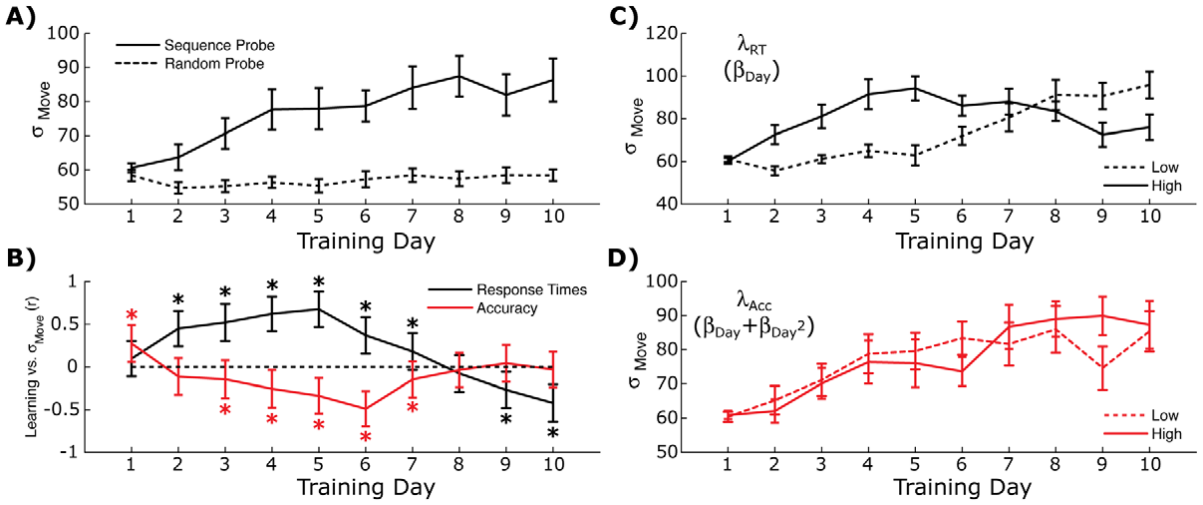
Relationship between response variability and learning rates. A) Variability of response times for both probe blocks. Errorbars show standard error across subjects. B) Correlations between individual subject learning rates, λ_RT_ and λ_Acc_, and reaction time variability on each day. Errorbars show standard deviation from bootstrap simulations (1000 iterations). Asterisks show significant one-sample t-tests (from a null of r = 0), after adjusting for multiple comparisons (Bonferroni correction for 10 comparisons). C) Response time variability during the Sequence Probe block for high and low response time learners. Errorbars show standard error across subjects. D) Same as *C* but with subjects separated by accuracy learning rates.

Computational models of sensorimotor control [42], [43] and animal models of sequential learning [37], [38] suggest that variability in the planning process can play a critical role during learning. We looked at how variability in response times changed across the training period in both the Random and Sequence Probe blocks (Figure 3a). In the Sequence condition, we found a consistent increase in movement variability across all training days (repeated measures F(22,207)  = 4.50, p<0.0001). This increase in variability appeared to be selective to the sequence condition, since no such change was detected in the Random probe (dashed lines in Figure 3a; repeated measures F(22, 207)  = 1.18, p = 0.309).

If this expanded response variability was related to the across-day learning rates, then individual differences in response variability should predict individual differences in learning rates, i.e., does more variability on early training days correlate with faster improvements in response times across the entire training period? To test this we correlated each day's response time variability with the across-day learning rates for each subject (standard deviations were estimated using a bootstrap with 1000 iterations, [44]). During the early phase of training (Days 2–7), movement variability had a strong positive correlation with overall response learning (black lines, Figure 3b). As training progressed (Days 9–10), response variability went from being advantageous for reaction time learning, to being counter-productive. An opposite, albeit weaker, pattern was observed for the relationship between response variability and accuracy learning rates (red lines, Figure 3b). At the early phase of learning (Days 3–7), greater variability correlated with less accuracy learning. At later stages of learning, reaction time variability had no relationship with across day learning rates for accuracy.

This pattern of correlations between response variability and learning suggests that optimal learners expand their movement variability early in the learning process (i.e., before Day 8), albeit with a slight cost to accuracy, and contract variability at later training days. To explicitly test this, we categorized subjects as being either high or low learners on both λ_RT_ and λ_Acc_ separately. This categorization was done by performing a median split on the learning rate scores. Consistent with the lack of correlation between λ_RT_ and λ_Acc_, only 39% of our participants were found to be overall high or low learners (i.e., have high λ_RT_ and high λ_Acc_ scores or have low λ_RT_ and low λ_Acc_ scores). The remaining subjects were evenly split between a primarily speed-based learning strategy (i.e., high λ_RT_ and low λ_Acc_, 30.4%) or an accuracy-based strategy (i.e., low λ_RT_ and high λ_Acc_, 30.4%).

For each learning type (λ_RT_ or λ_Acc_) we looked at how high and low learners modulated their reaction time variability across training. Consistent with our predictions, for high versus low λ_RT_ participants, we found that the better learners showed an initial rise in movement variability during the early phase of training (Days 2–6) followed by a sharp drop in variability on later training days (solid lines, Figure 3c). In contrast, low λ_RT_ learners showed a monotonic increase in movement variability across training. This between-group difference in movement variability was not found when participants were split based on λ_Acc_ scores (Figure 3d), although the mean for the high learners was slightly lower than the mean for the low learners during the early phases of learning as predicted by Figure 3b. These findings suggest an advantageous learning strategy, at least for response speed, that involves expanding the variability of planned responses during early stages of training followed by reduced movement variability as sequence structure crystallizes.

### Emergence of the post-error slowing

**Figure 4:**
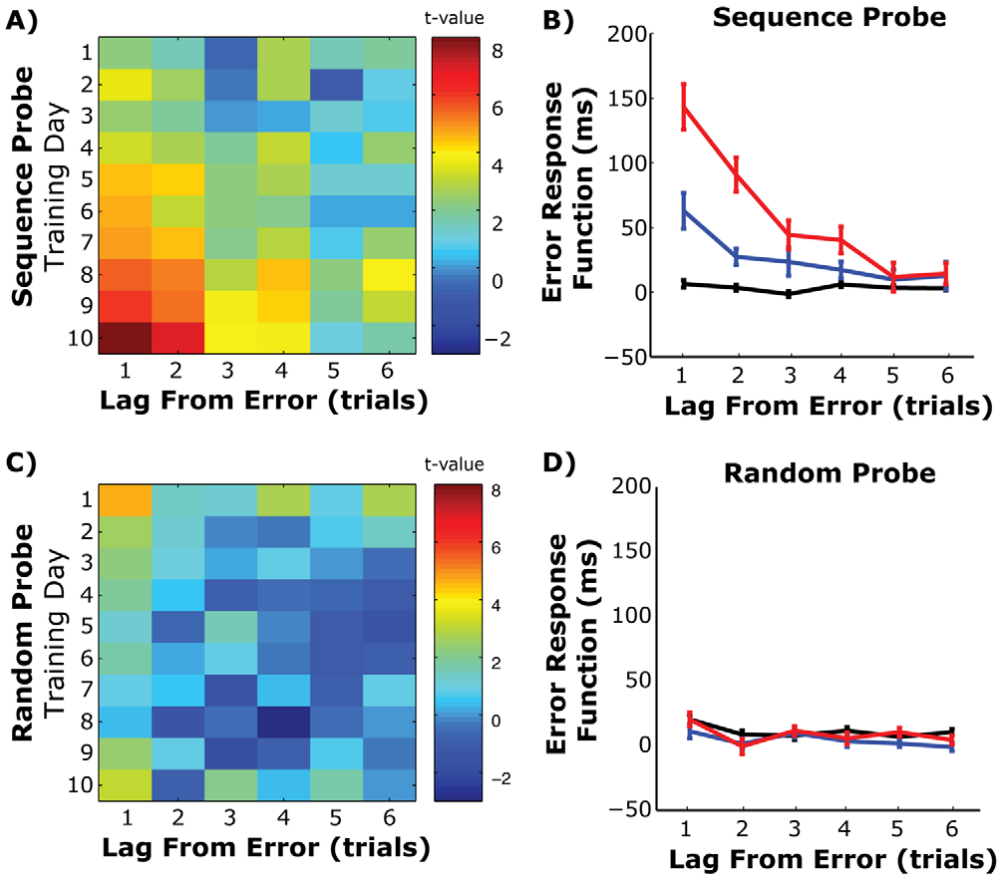
Emergence of post-error slowing with training. A) Post-error slowing shown using the error response function (ERN) for 6 trials after an error and each training day. Data are shown as one-sample t-tests across subjects. B) Mean and standard error ERN across subjects for the first (Day 1), middle (Day 5) and last (Day 10) day of training. Each value shows the average response time, in milliseconds, at six different lags relative to the block mean value for that day. C) Same as *A* for responses during the Random probe block. D) Same as *B*, but for Random probe block trials.

As skills become consolidated, particularly cognitive skills that monitor performance, the presence of an error can introduce a characteristic slowing of subsequent trials [39]. This effect is sometimes referred to as a post-error slowing and may reflect either conflict monitoring [40] or reorienting processes [41]. This effect could also be consistent with a response-binding hypothesis, since subsequent responses are no longer independent motor plans but part of a larger meta-motor plan and disrupting the motor plan may carry over to subsequent behaviors. To measure this effect across subjects, we calculated the average reaction time across subjects and training sessions after an error (Figure 4). This is referred to as the error response function (ERF). During the Sequence probe block, errors had increasing influence on subsequent response times (Figure 4a). This is shown as both a main effect of training day (repeated measures F(9,990)  = 15.42, p<0.001) and a Day-by-Lag interaction (repeated measures F(45,990)  = 6.54, p<0.001) in the ERF. By the end of training, a single error could delay response times of up to 4 trials (Figure 4b). This effect only occurs during the Sequence probe block, as no such pattern is present when the same analysis is performed on responses during the Random probe condition (Figure 4c,d), and there is no significant Day-by-Lag interaction on the ERF (repeated measures F(45,990) <1). Thus, as with higher-level cognitive skills, we also see evidence of post-error slowing in our sensorimotor sequence learning task.

### Response binding

Neural models of sequence learning suggest that learning happens by binding independent responses together in a unified action plan [15], [45]. Typical approaches to measuring response chunking in the context of the SRT involve looking for signatures of response boundaries in the response times [13], [16]–[22], [26]. The logic of this approach is that, if a set of items is bound together and separated from a second adjacent set, then the first item in a chunked sequence of responses will be significantly slower than the rest of the items in the set [21]. Such an approach is simple when dealing with small and specifically constructed sequences so as to highlight easy-to-define chunk boundaries. However, the complexity of the sequence used here precludes this style of analysis because it was constructed so as to minimize obvious structures that would facilitate detection of the sequence (see Methods).

**Figure 5:**
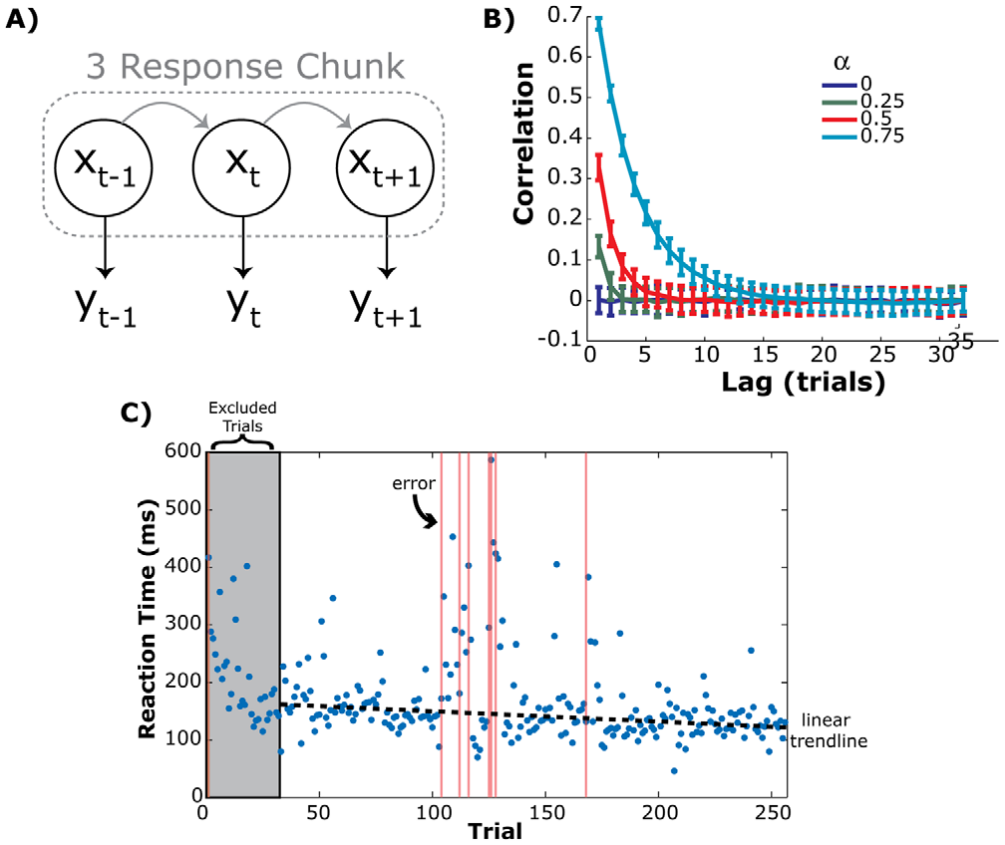
Outline and predictions of the response binding hypothesis. A) Before learning, the internal plan (x) on each trial (*t*) is temporally independent as is each response time (y). With training (gray lines) each command becomes dependent on the properties of the previous trial, forming a chunk. B) Autocorrelation functions for simulated trials using the model shown in *A* and different inter-plans binding terms (α). C) Example Sequence probe block from a single subject (same data as Figure 1B). Each blue dot shows a single trial reaction time. The gray window shows the first cycle of the sequence that was excluded from the autocorrelation analysis. The dashed black line shows the linear trend line that was removed before autocorrelation analysis. Each vertical red line shows an error trial.



Therefore, we developed a novel analytical approach based on two assumptions: 1) as internal response plans become coupled (i.e., dependent) their output should become more correlated, 2) execution/motor noise is independent across trials. We can think of the responses across trials as a chain of random variables consisting of internal plans (x) and observable responses (y), where 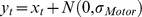 (Fig 5a). Probability theory holds that the joint probability of any two responses is

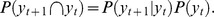



Before learning, each plan is independent from another, i.e.,
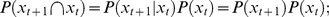
however, as two temporally adjacent plans become bound together 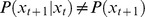 and the properties of the two responses become correlated. Since y_t+1_ is defined by x_t+1_, the resulting output should also exhibit this dependency. This property extends across multiple responses according to the chain rule, such that



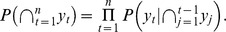



Therefore multiple key presses that reflect a shared internal command should exhibit an increased correlation in their trial-by-trial responses.

If this is model is true, then adjacent responses should become correlated with each other across training in the Sequence Probe condition. To illustrate this we simulated a simple motor planning system where on each trial the response plan is estimated as 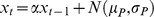, and the motor output is 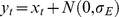. In this simulation we set µ_P_  = 200 ms, σ_P_  = 10 ms, and σ_E_  = 10 ms. We then tested a range of inter-trial binding parameters, α, from statistically independent across trials (α  = 0) to strongly coupled (α  = 0.75). For each binding parameter, we ran a set of 100 simulated blocks, with 1000 simulated trials per block.

**Figure 6:**
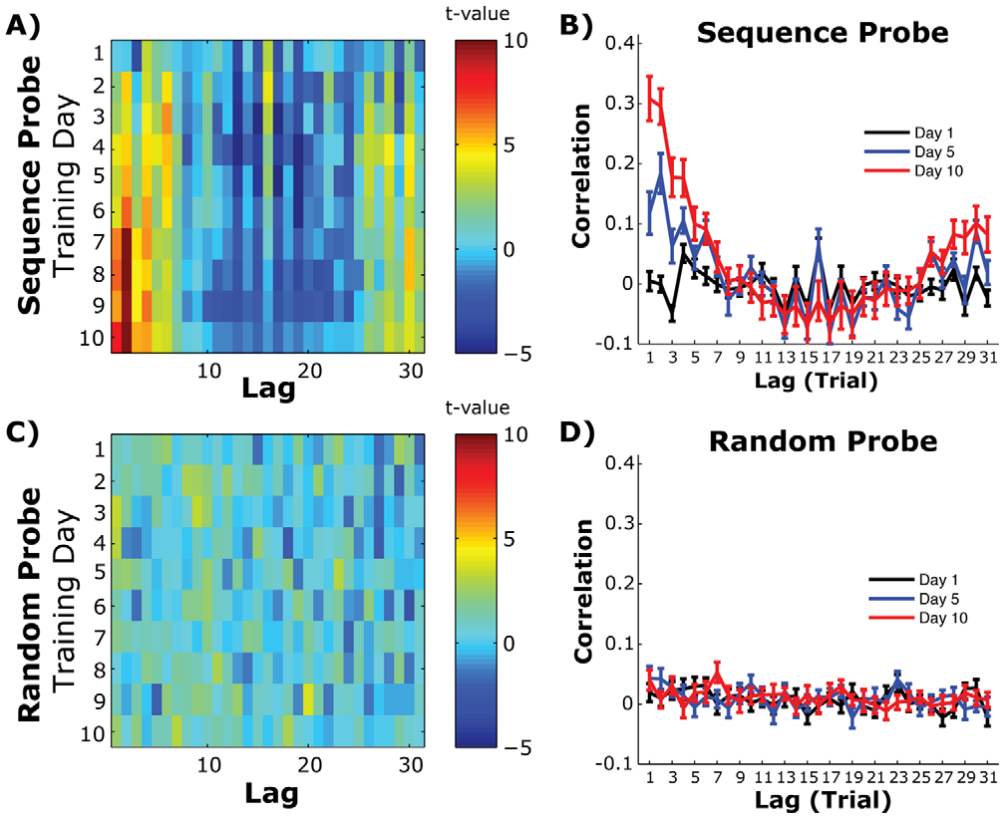
Autocorrelation of response times across the training period. A) Heatmap of the autocorrelation functions for each training day and lag value. Data is presented as one-sample t-test values across subjects. B) Mean and standard error autocorrelation functions, across subjects, for the first (Day 1), middle (day 5) and last day of training (Day 10). C) Same as *A* for responses during the Random probe block. D) Same as *B*, but for Random probe block trials.

Figure 5b shows the autocorrelation function for the simulated responses, y, from this simulated experiment. As the binding parameter between internal plans gets stronger we see a peak emerge in the autocorrelation function. This peak in the response time correlation function can then be used as an index of response chunking across plans. To specifically test this approach, we isolated the linear component of the Sequence probe block (Figure 5c), removed the slow linear trend, and looked at the autocorrelation of the residual response time vector. Consistent with the binding hypothesis, we detected a consistent pattern emerging in the autocorrelation function across training days in the Sequence probe block (Figure 6a,b). A two way repeated measures ANOVA detected a significant Day-by-Lag interaction (F(288,6336)  = 3.44, p<0.001), consistent with a learned peak in the autocorrelation function across training. This effect appears to be mainly expressed during the Sequence probe block. An identical analysis performed on the preceding Random probe block found a much smaller interaction (Figure 6c,d; Day x Lag interaction F(288,6336)  = 1.16, p = 0.035) however this comparison did not pass significance after a Bonferroni correction for multiple comparisons (adjusted p = 0.025).

**Figure 7:**
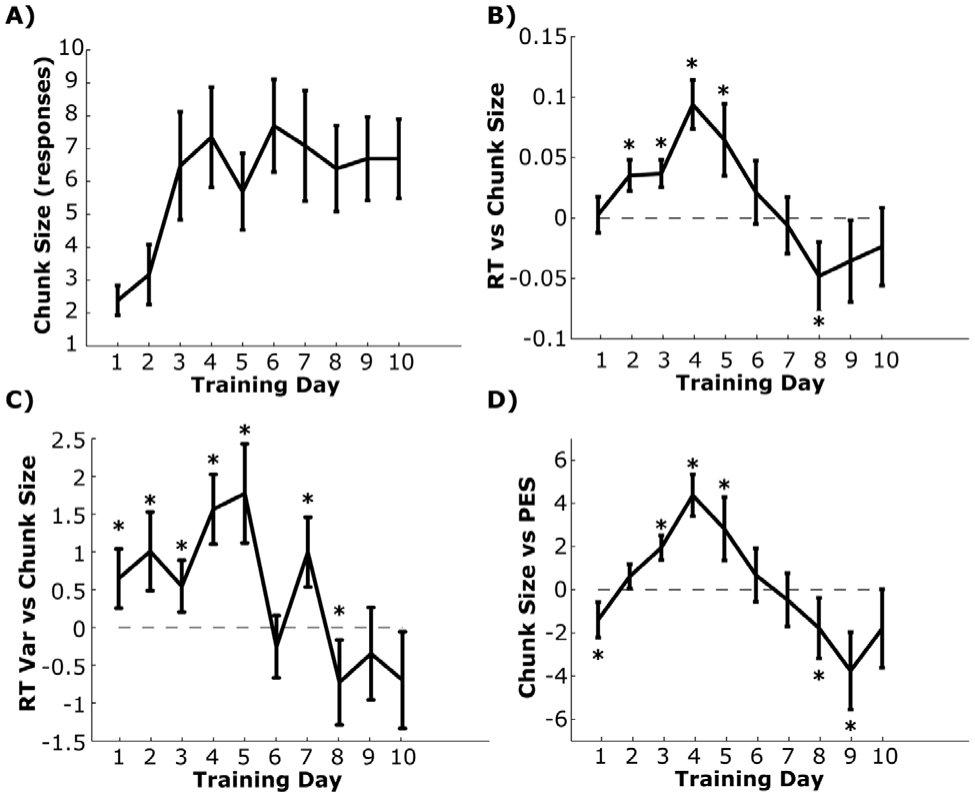
Estimated chunk size and relationship to other behavioral changes. A) Mean and standard error of estimated chunk size based on the autocorrelation for each day. B) Regression values for the relationship between chunk size and mean response times. Error bars show the adjusted 95% confidence intervals generated using a bootstrap method and after correcting for 10 comparisons. Asterisks show significant effects. Dashed line shows the null mean (i.e., 0). C) Same analysis as *B* but for chunk size and response time variability. D) Same analysis as *B*, but for the post-error slowing (PES) effect using the first lag after an error as an index PES.

When we isolate the autocorrelation function in the Sequence Probe for the first, middle (Day 5) and last (Day 10) training day, we see significant correlations extending out to 7 lags by the end of training, suggesting that the response time on one trial significantly predicts the response time 7 trials later (Figure 6b). In fact, we can use the number of statistically significant lags from lag = 1 as an index of set size in the bound sequence (see Methods). We found that the hill in the autocorrelation function asymptotes at the end of the first week of training at ∼7 lags (Figure 7a; repeated measures F(22,207)  = 2.011, p = 0.04).

It is common in SRT studies to assume that learning related improvements in overall response time are related to response chunking, i.e., actions get faster because response plans are getting bound together. If this is true, then we should see a correlation between the size of a response chunk and sequence specific response times. We tested this using bootstrapped regression to model the relationship between chunk size and response time scores on each day. While overall response times correlated with chunk set sizes early in learning (Days 2–5), this relationship disappears on later training days once chunk size asymptotes (Figure 7b). This utility early in learning resembles patterns seen in the relationship between response variability and across-day learning rates (see Figure 3). So we ran the same analysis using response time variability on each day as the dependent variable. As expected we also see a similar phasic relationship across the training period, albeit much noisier than when average response times are used as the dependent variable (Figure 7c). Therefore, both the mean and variability of response times are only associated with the size of a chunked set early in training.

Finally, as mentioned in the previous section (Results, *Emergence of the post-error slowing*), the presence of a post-error slowing might reflect the consequence of disrupting a bound sequence of responses. If this is correct, then we should see a consistent correlation between the magnitude of the post-error slowing and size of the chunked set. To test this, we took the average response time after an error for each subject and each day and correlated these values against chunk sizes on that day. This correlation pattern was nearly identical to that observed with the mean response times, where the significantly positive associations on Days 3–5 of training.

### Trial-by-trial dynamics of learning

The autocorrelation in response times and the increased post-error slowing with training are consistent with a response-binding hypothesis with learning. The conceptual model in the previous section (Results, *Response biding*) posits only a dependency between adjacent response plans. However, there are many possible features from which a response planning system can learn [46]. Therefore, to come up with a conceptual understanding of the underlying dynamics that link consecutive trials together, we adopted a state-space modeling approach that simulates the trial-by-trial dynamics of the response preparation process (see Methods, *State-space model*). This model fits in a class of state-space models of internal response planning [7], [42], [43], [47]–[49] rather than the dynamical control models used to explain repetitive or oscillatory behavior [50].

**Figure 8:**
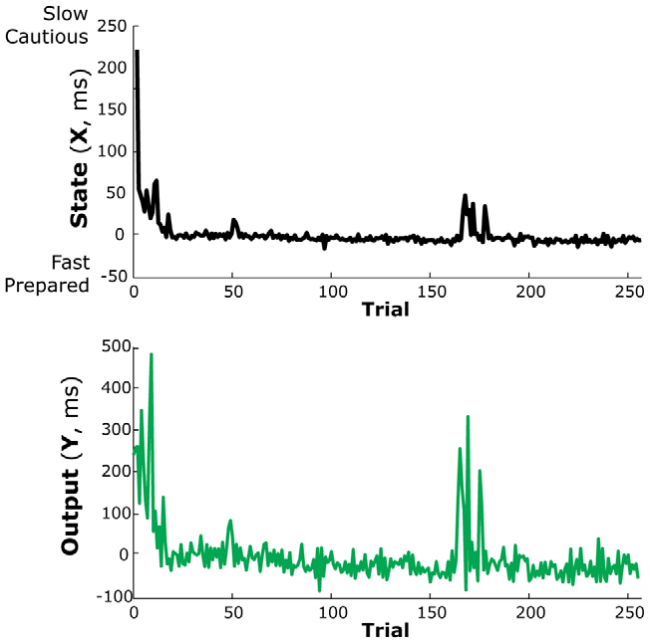
The simulated response state (top panel) and output values (bottom panel) during the Sequence probe block for an example subject at the beginning of training. Negative states reflect a system prepared to make a faster output response. Positive states reflect a slower, more cautious system.

Using the time-series of reaction times and the error-feedback signal on each trial, we modeled the dynamics of a simulated response preparation state, *X_t_* (Methods, Eq. 1), and the adaptive response dynamics of the behavioral output, *Y_t_* (Methods, Eq. 2). This reflects a more sophisticated model than the qualitative Markov-Chain model used above (see Results, *Response binding*). An example simulation during a Sequence probe block for one subject is shown in Figure 8. Normally these types of dynamic models are used to model the sensorimotor system's adaptive dynamics to learn a new mean output after a perturbation [42], [47], [48]. Here, we used this dynamical systems approach to model how the system learns to be more or less prepared to make the subsequent response.

**Figure 9:**
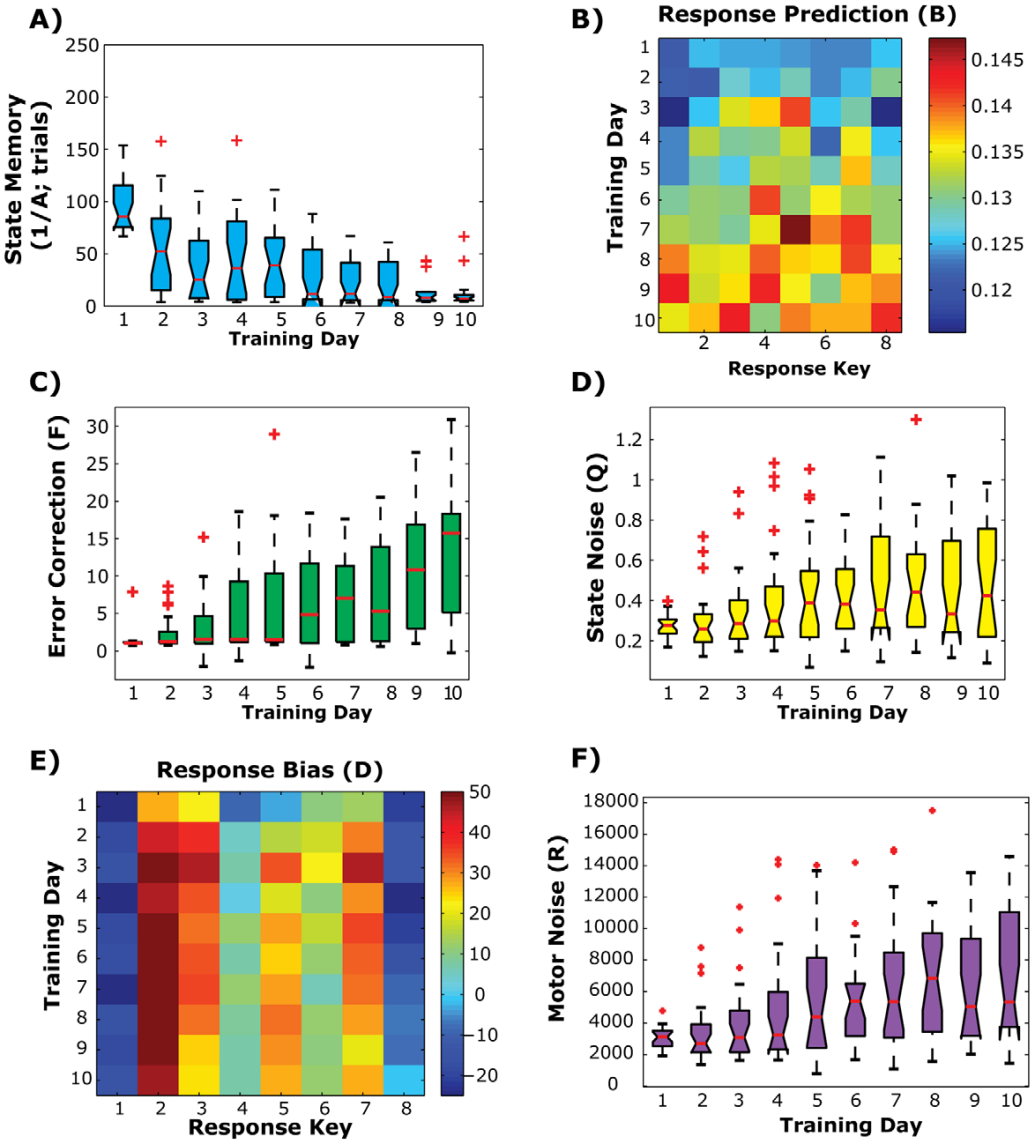
State-space model results. A) Whisker plots showing the change in the fitted state memory term (A) across training days. Data presented as inverse parameter values to reflect time to change state (in trials). Same plotting conventions as Figure 2a,b. B) Heat map of average response prediction term (B) across training. C,D) Whisker plots of the error correction term (F) and state noise term (Q, in variance units) respectively. Same plotting conventions as *A*. E) Heat map of the average response bias term (D) from the output equation (Methods, Eq. 2). F) Whisker plot of the motor noise term (R, in variance units) across training day. Same plotting conventions as *A*.

Figure 8 shows the free parameters of this state space model across training. In the state update part of the model (Methods, Eq. 1), there are four parameters: state memory (A), response prediction (B), error correction (F), and state noise (Q). The first term we looked at was the state memory parameter (A), which measures the degree to which the previous state on trial, *t*, influences the subsequent trial, *t+1*. This is analogous to the binding parameter, α, used in the previous model (see Results, *Response binding*). With training, the state memory term gets stronger (repeated measures F(22, 207)  = 5.86, p<0.001; Figure 9a), meaning that the previous state has a much stronger influence on the preparedness of the following trial. The asymptote in state memory values means that by the last day of training it only takes a few trials for subjects to transition between fast and slow states because the previous state has a much stronger influence on subsequent response plans.

The response prediction term (B) captures the degree to which a response at a single key on one trial influences the preparedness for making a response on the subsequent trial. So, rather than having preparedness states being associated across trials, the executed action influences subsequent preparedness states. For example, how does making a fast response with the left index finger make a subject more or less prepared to make a response on the next trial? This parameter also showed a strong training effect, illustrated by a significant main effect of training day (Figure 9b; repeated measures F(9,1386)  = 4.47, p<0.0001). This means that the influence of a single key press on the subsequent response state was stronger with longer training. This effect appeared to be expressed equally for all response keys, since we did not detect a significant main effect of response key (repeated measures F(7,1386)  = 2.07, p = 0.050) and only a small day by response key interaction (repeated measures F(63,1386)  = 1.60, p = 0.0024, Bonferroni corrected threshold p = 0.0084). Therefore, consistent with the increased association of adjacent responses during the Sequence probe trial, we see an increased internal prediction of future response states with training. Being faster on one trial means that subjects were faster on the next trial, while being slower on a trial slowed down the following trial response.

The error corrective term (F) measures the influence that committing an error on one trial has on the preparedness to make a response on the following trial. This parameter should reflect the degree of post-error slowing. As expected, F also showed a strong training effect (Figure 9c; repeated measures F(22,207) = 12.30, p<0.01). In this case, the presence of an error on one trial slowed down the response state. By the end of training a single error could delay the internal state response by ∼15 ms. This effect gets compounded across trials because of the increased state memory (A), likely reflecting the multi-trial effect seen in the ERF (Figure 7).

The state memory, response prediction and error-corrective parameters are the terms in the state model that best capture learning related changes on a trial-by-trial basis. We wanted to see whether changes in these terms with training were correlated or learned independently. To simplify analysis, we created a composite response prediction score for the first and last day of training by averaging across all response key values. Learning changes were assessed by subtracting scores on the first day of training (Day 1) from the last day of training (Day 10). All three scores showed highly significant learning effects (A: t(22)  = 7.93, p<0.001; B: t(22)  = 5.45, p<0.001; F: t(22)  = 6.17, p<0.001), however we did not detect a significant correlation between changes in A & B (Spearmans r  = −0.27, p = 0.09), A & F (Spearmans r = 0.07, p = 0.348), or F & B (Spearmans r = 0.11, p = 0.313),. Thus changes in state memory, response prediction component, and the error-corrective component appear to occur at different rates.

Finally the state noise term (Q) estimates the internal variability in the response planning system. This parameter also showed significant training effects, such that later training sessions had more noise (Figure 9d; repeated measures F(22,207)  = 3.64, p<0.01). While at first this may seem antithetical to increased learning, it has been well established that state noise is correlated with increased state learning [43]. In this way, feedback learning results in a more energetic internal state that also increases the noise of the planning system. This pattern also follows the overall change in reaction time variability that we observed (Figure 3a).

In contrast to the internal state, the response bias term (D) in the output portion of the model failed to show slightly less consistent learning effects (Figure 9d). This term reflects the inherent output bias for each finger; i.e., is the left pinky faster than the right index finger? While there was a significant main effect of training day (repeated measures F(9,1386)  = 3.54, p = 0.0004), there was a much stronger main effect of response key (repeated measures F(7,1386)  = 55.78, p<0.0001). The training day by response key interaction was also significant (repeated measures F(63, 1386)  = 2.28, p<0.001). However, most of the training effects appeared to stem from changes that occurred after the initial training session (Day 1). If this day is removed from analysis, we still observed a very strong main effect of response key (repeated measures F(7,1386)  = 54.75, p<0.0001), however the main effect of training day disappears (repeated measures F(8, 1386)  = 1.21, p = 0.30). Post-hoc tests reveal that the main effect of response key is driven by faster responses with the pinky fingers and index fingers than the ring or middle finger. So while there is a consistent response bias such that certain key presses are easier to execute than others, there was no effect of training on the simple motor execution. This is consistent with the lack of training effects observed during the Random probe condition.

While the response bias term failed to show significant training effects, we did see a steady increase in motor noise (R) across training sessions (Figure 9e; repeated measures F(22,207)  = 6.46, p<0.0001). This term shows the noise in the motor plant that executes an action. Similar to the state noise term, the motor noise term increased with training, suggesting a more variable output with training. Again, this is likely a bi-product of the increased trial-by-trial dynamics of the internal state and its carry-over to downstream execution processes as has been shown in previous studies [43], [47].

## Discussion

We found that prolonged training on a complex, bimanual SRT paradigm results in continued behavioral improvements across two weeks of training. Based on the post-hoc questionnaire, subjects transitioned from being unaware of the sequence to being aware of its presence on certain blocks of trials after 5 days of training. This coincided with an asymptote of accuracy rates, but not response times. Indeed, our measure of sequence specific response time changes showed continued improvement up to the last day of training. Analysis of across-session learning rates suggests that response speed and response accuracy are learned independently. For learning of response speed, an optimal performance strategy appears to be to quickly expand response time variability during early phases of learning and then contract this variability at later stages of learning. Consistent with a response binding hypothesis [15], [21], we observed the emergence of both a post-error slowing [39] and increased correlation in response times across trials. The size of the chunked set asymptotes at ∼7 trials after the first week of training, despite continued improvements in response speeds into the second week of training. Using a state-space model of the internal planning processes, we confirmed that as learning crystallizes, subjects exhibit greater dependency between the plan on one trial and the plan on subsequent trials (i.e., increased state memory) as well as the ability to both predict future responses and exhibit a greater penalty in subsequent response times for committing an error. Taken together, these results show how complex sequential skill learning is a highly dynamic process at long timescales of training, with multiple component processes that lead to performance improvements at different stages of the training process.

Evidence of response binding during sequence learning has previously been demonstrated within shorter training sessions[13], [14], [16]–[22], [25], [26]. Koch and Hoffmann (2000) showed how chunking is partially based on relational patterns in the stimulus presentation itself. For example, the triplet “1-3-1” is an easy three response pattern to both consciously detect and bind as a unified response. Our sequence was constructed to minimize these types of easy to detect cues. First, we extended the sequence length to be much longer than the typical pattern used in an SRT task. Second, we started each sequence block at a random position in the list, so as to not always begin each block of trials with the same key presses. Finally, we explicitly generated a sequence pattern that did not contain any triplet structures so as to minimize observable relational patterns. This is likely why the post-hoc questionnaire showed that subjects were unaware of a sequential ordering of the stimuli until after several days of training, rather than within the first or second training session. However, despite these measures several aspects of the task encouraged explicit awareness. The direct error signals, the discrete blocking of Random and Sequence trials, and even the post-hoc questionnaire itself could all serve as distinct clues to the presence of the sequence. So while our definition for moving from implicit to explicit strategies was the ability to detect a pattern of cues on specific blocks of trials (i.e., a questionnaire score of 2), this is in fact a fairly conservative threshold. It is entirely possible that simply being aware of the presence of a pattern may engage more explicit learning systems. In addition, as skills become automatic, there is a return to an implicit strategy during the execution process (for review see [3]). Since we did not test for automaticity (e.g., adopt a dual task probe), we cannot be sure if this return to implicit strategies took place within the 10 day training period. Future studies should focus on disentangling verbal awareness from automaticity with long-term training, in order to better understand the dynamics that lead to response binding during skill learning.

Based on the autocorrelation results (Figures 6 & 7a), by the end of training, up to seven discrete key presses had become correlated across time. This item length is particularly interesting given its similarity to the 7+/− 2 item limit of the working memory in the classic digit span measure [51]. Indeed, working memory appears to be critical for general sequence learning [29] and correlates with the magnitude of chunk boundaries using more traditional chunk estimation methods [30].While this may suggest a strong working memory component in the high-level explicit learning of the sequence structure, it should be pointed out that the autocorrelation functions in our sample did not appear to asymptote by the end of training. Thus further training may broaden this item span beyond the “magical” 7+/− 2 length. However, it should be pointed out that even training on the digit span task can dramatically increase the working memory span length as well [52].

Although response chunking is assumed to be a key mechanism in response time improvement, we only detected correlations between chunk size and overall response speed in the Sequence Probe blocks during the early training days (Figure 7b–d). At first, this pattern of results may suggest a transitional relationship between chunk size and response times. However, this pattern may also indicate a lack of true relationship between these variables. Unlike the movement variability results shown in Figure 3, chunk size has a clear asymptote at the end of the first week of training. If chunk size and response times (or post-error slowing) are linked through a common third variable, but not directly to each other, then any correlation between these variables would quickly disappear once the chunk sizes asymptote. Without more sophisticated analysis, we cannot rule out this possibility when interpreting these results.

The post-error slowing with training is also particularly interesting because this is typically interpreted as the result of conflict monitoring [40] or reorienting processes [41]. While we interpret our findings in the framework of a response-binding hypothesis, we cannot rule out the possibility that this slowing reflects a high-level monitoring of errors. In fact, error-corrective learning is thought to be a key component of the sequence learning process (see [3] for a review). Conflict models of post-error slowing propose that this arises from monitoring of planning errors, for example, when you make a plan to hit the “k” key on a computer keyboard but it is not the appropriate character for the word you are typing (as opposed to execution errors like planning to press the “k” key but seeing “j” appear on the screen instead). The monitoring of planning errors is thought to be mediated primarily by connections between the anterior cingulate gyrus and the basal ganglia [40], the latter of which is also known to be critical for long-term skill learning such as sequence learning [12]. Future research is needed to explicitly disentangle conflict monitoring, error correction and response binding.

The state-space model was intended to be a first pass at disentangling portions of these component processes. Consistent with typical state-space models of motor learning (see [46]), our results suggest that over time subjects learn a forward model of future response plans based on the previous trial responses. Being faster on individual trials (i.e., more prepared), means that subjects are more likely to be even faster on following trials. This inter-response dependency likely interacts with the increasingly dynamic state memory (A) as well, in order to bind adjacent response plans together. The increased state memory with training also suggests strong response “chunking” by showing how individual response plans become more dependent over time. The initially slow memory rate (i.e., requiring many more trials to reset the state) at the beginning of training is likely a precaution against learning on noise. As subjects become more certain of future response cues, it becomes necessary for the response preparation state on one trial to influence the next, thus resulting in a more dynamic internal state. As the state and learning dynamics increase, so does the noise of the system (i.e., state noise, Q, and motor noise, R) due in large part to the increased energy of the system [43].

It is important to point out that, in this study, response speed and error-corrective mechanisms appear to be learned independently. Our first evidence for this comes from the observation that reaction time learning rates and accuracy learning rates are uncorrelated in this sample. This is bolstered by the observation that changes in the response prediction component and the error-corrective component of the internal state model also occurred at different rates. Taken together these findings highlight the multiple mechanisms at play when learning to bind multiple responses into a unified sequence of actions.

It should be noted that our measure of response binding is dramatically different than previous measures of response chunking [13], [14], [16]–[24], [26]. In nearly all of these studies, the slowing of responses to one or two items in the sequence is used as an index of the start of a new set. Our approach assumes that as temporally adjacent plans get bound together, this will be reflected in the autocorrelation function of response times. While it has intuitive appeal, this model is inherently incapable of determining whether chunks vary in size across the sequence, isolating segmented boundaries between chunks, or estimates of the strength or degree of chunking. Clustering based approaches have recently shown promise at characterizing complex sequence patterns [25]. Future work should look to improve the current methodological approach by finding ways to characterize specific chunk boundaries on such long and complex sequences.

There is ample evidence to suggest that the response chunking observed in this study is likely to be dependent upon cortico-basal ganglia systems. First, recent functional imaging experiments have show how novel measures of chunk concatenation in a similar task correlates with activity in a frontal-striatal network [25]; although this study only looked at changes across a few days of training. In our study, the emergence of the auto-correlation and error-related delays in response times occurred after several days of training. This time-scale is consistent with the time-scale of learning mediated by corticostriatal systems [53]. Second, there is ample evidence from the neurophysiological literature that behavioral chunking depends on corticostriatal networks. In rodents, daily training on a T-Maze task shows that responses of striatal cells become time-locked to different stages of the task itself, suggesting the emergence of the sequence of behaviors necessary to complete the task [33]. Indeed, blocking of striatal dopamine activity with raclopride, a D2 receptor antagonist, interferes with the chunking of a new motor sequence in primates, but not the recall of an overly learned sequence pattern [23], [24], [32]. Indeed, this timescale of learning and the implication of basal ganglia pathways fits with current models of motor skill consolidation (see [3] for review).

Similar sequential learning processes are also found in the learning of social songs in song birds (for review see [54]). As mentioned in the Introduction, during development these birds learn to bind a sequence of vocalizations together into a stable and highly stereotyped song. It is well established that the AFP, the bird-song analogue to the cortico-basal ganglia system, is critical for this learning process, but not for the execution of an already learned song. One interesting observation from this literature is that, during early stages of learning, juvenile males will modulate the variability and precision of their “motifs” depending on the presence (directed song) or absence (undirected song) of a female [35]–[37]. Much of this variability during undirected songs is driven by variability in the firing dynamics of AFP neurons [35]–[38]. It is believed that this expanded variability is critical to the learning process as a way of exploring the space of possible songs (i.e., as a way of practicing slight variations when not trying to impress a female). As a song crystallizes into a stereotyped vocalization, this modulation of variability in undirected contexts disappears. In our study, we found a similar dynamic modulation of response variability during learning. Overall response time variability increased with training. However response variability was a predictor of better learning only during early phases of training and became detrimental to learning at later training days. In fact, better learners, at least in terms of learning response speeds, expanded response time variability more during the early phase of learning and then contracted it on later phases of learning. Thus, while response time variability is often seen as “noise” from sensorimotor systems, this finding highlights a possible utility for variability in the learning process itself.

Finally, in humans it has long been known that the basal ganglia are critical for novel behavioral patterns to become automatically programmed skills [12], [55]. This type of automaticity is typically measured using a dual-task probe condition. Unfortunately, as mentioned above, we did not test the automaticity of the learned sequences across training to see if patterns go from being interfered by the dual task to being unaffected by the dual task. Future work should also focus on how these response dynamics change with degree of automaticity, in order to confirm the likelihood of the cortico-basal ganglia system being involved in the learning process, as well as the degree of consolidation of the learned sequence itself.
